# Trends in Pit and Fissure Sealant Use and Decayed, Missing, and Filled Teeth Rates Among Korean Adolescents After Including Dental Sealants Under Insurance Coverage

**DOI:** 10.1111/jphd.70052

**Published:** 2026-03-10

**Authors:** Mina Kim, So‐Jung Mun, Sun‐Young Han, Han‐Na Kim, Jung Yun Kang, Hiejin Noh

**Affiliations:** ^1^ Department of Dental Hygiene, The Graduate School Yonsei University Wonju Gangwon‐do South Korea; ^2^ Department of Dental Hygiene, College of Software and Digital Healthcare Convergence Yonsei University Wonju Gangwon‐do South Korea

**Keywords:** adolescent, dental sealant under insurance coverage, DM, FT rate, joinpoint regression analysis, Korean National Health and Nutrition Examination Survey, Korean Youth Risk Behavior Survey, pit and fissure sealant

## Abstract

**Objectives:**

This study examined the effects of dental sealant insurance coverage on trends in pit and fissure sealant (PFS) use, decayed, missing, and filled teeth (DMFT) rates, and filled teeth (FT) rates among Korean adolescents, using data from the Korean Youth Risk Behavior Survey (2005–2024) and the Korean National Health and Nutrition Examination Survey (2007–2015).

**Methods:**

Data from participants aged 12–18 years were analyzed. The proportion of PFS use referred to the proportion of respondents who answered “yes” to the question “Have you received PFS in the past 12 months?” in the survey. DMFT and FT were classified based on oral examination criteria from 2007 to 2015. Statistical analyses were conducted using Joinpoint regression (version 4.8.0.1) and PROC SURVEYLOGISTIC in SAS 9.4 to estimate the annual percent change (APC), odds ratios, and 95% confidence intervals.

**Results:**

PFS use increased until 2018 (APC = +1.5%) and declined slightly thereafter until 2024, whereas DMFT and FT rates decreased from 2007 to 2015. Dental sealant insurance coverage was significantly associated with higher PFS use and lower DMFT and FT rates (*p* < 0.05).

**Conclusions:**

Dental sealant insurance coverage effectively improved oral health and reduced dental caries among Korean adolescents.

## 1 Introduction

The World Health Organization recommends the use of pit and fissure sealants (PFS) to prevent dental caries in children and adolescents [1]. According to a systematic literature review, an analysis of 38 randomized controlled trials conducted with 7924 children and adolescents showed that PFS is effective in preventing dental caries on the occlusal surfaces of permanent teeth. As such, PFS has been reported to be an effective method for preventing dental caries [2].

Several countries have implemented PFS policies to prevent dental caries in adolescents. Similar to the United States, which provides PFS through Medicaid and the Children's Health Insurance Program [3], Canada offers PFS through the Healthy Smiles Ontario program, reporting that 1440 children received public health dental services in 2022, many of whom received preventive services, including PFS [4, 5].

The Ministry of Health and Welfare of South Korea initiated a PFS oral health project for children in 2002 [6], and included PFS for the first molar without dental caries in children aged 6–14 years under dental sealant insurance coverage on December 1, 2009. In 2010, the scope of dental sealant insurance coverage was specified as the occlusal surface. After PFS was included under dental sealant insurance coverage, the prevalence of dental caries in children decreased. However, considering cases where dental caries were not covered due to age—particularly among children under 6 years who developed their first molars early—the coverage age range was expanded from 6–14 years to < 6 years on October 1, 2012. The second molar was also covered by insurance, given its high probability of developing dental caries within 1 year, similar to the first molar. Thereafter, the target age range continued to expand. On May 6, 2013, coverage was expanded to include children < 18 years of age. In 2017, the out‐of‐pocket rate was reduced from 30%–60% to 10% [7, 8, 9, 10]. Most existing studies that examined the effectiveness of these policies analyzed short‐term trends in PFS use, decayed, missing, and filled teeth (DMFT), and filled teeth (FT) rates [11, 12].

Shin et al. compared PFS use rates in 2007–2009 (before policy implementation) and 2013–2015 (after policy implementation) to evaluate changes following the inclusion of dental sealants under insurance coverage in 2009. The results indicated that PFS use rates among children aged 12–18 years were significantly higher in households with higher incomes and among those who received regular oral checkups [11]. In addition, a study by Choi et al. reported that the proportion of PFS use increased from 27.8% to 35.5%, whereas DMFT rates decreased from 68.4% to 59.3% among policy beneficiaries, unlike in nonbeneficiaries [12]. Given that most existing studies analyzed only limited time periods, the present study aimed to identify the relationships between dental sealant insurance coverage and the proportions of PFS use, DMFT rates, and FT rates from 2005 to 2024 through long‐term analysis.

## 2 Methods

### 2.1 Data Collection and Participants

This study used raw data from the Korean Youth Risk Behavior Survey (KYRBS) (2005–2024) and the Korean National Health and Nutrition Examination Survey (KNHANES) (2007–2015) [13, 14]. The KYRBS selected approximately 56,000–79,000 samples from a population of approximately 2,581,000–3,930,000 middle and high school students from 2005 to 2024 using a stratified cluster sampling method. Of these, approximately 51,000–75,000 were included as study participants after excluding long‐term absentees, students with disabilities, and students with dyslexia (Table S1). The KNHANES participants were aged 12–18 years, and the sample sizes were as follows: 425 in 2007, 900 in 2008, 1033 in 2009, 795 in 2010, 715 in 2011, 683 in 2012, 721 in 2013, 551 in 2014, and 583 in 2015. This study was approved by the Institutional Review Board of Yonsei University Mirae Campus (IRB reference number: 1041849‐202503‐BM‐036‐01).

### 2.2 Study Variables

The proportion of annual PFS use was defined as the proportion of respondents who answered “yes” to the question, “Have you received PFS in the past 12 months?” in the KYRBS (2005–2024).

In the KNHANES, based on oral examination criteria from 2007 to 2015, dental caries experience in permanent teeth was classified as “D (Decayed teeth), M (Missing teeth), and F (Filled teeth),” whereas those who had received restorative treatment due to dental caries in their permanent teeth were classified as individuals with “F (Filled teeth).” The DMFT rate was defined as the proportion of individuals who had experienced dental caries in their permanent teeth, whereas the FT rate was defined as the proportion of individuals with “F.”

Sex, grade, region, academic performance, self‐rated household economic status, father's education, and mother's education were included as control variables influencing adolescents' PFS use. In addition, sex, age, region, and household income were considered variables related to dental caries experience [15, 16].

Considering the five time points at which PFS was included under dental health insurance coverage [7, 8, 9, 10] and the survey periods of the KYRBS and KNHANES (Table S2), the study period was divided into two segments—before and after the policy implementation—to identify differences (Table S3).

### 2.3 Statistical Analysis

PROC SURVEYFREQ was conducted using SAS software (version 9.4; SAS Institute Inc., Cary, NC, USA). Annual trends were analyzed using Joinpoint regression (Joinpoint Regression Program, version 4.8.0.1), and the annual percentage change (APC) and 95% confidence interval (CI) are presented. The timing of PFS insurance policy implementation is indicated using vertical solid, dotted, dashed, dash‐single dotted, and dash‐double dotted lines. Graph visualization was performed using the ggplot2 package in R software (version 4.2.3, R Inc.). The relationship between PFS insurance coverage and the annual proportions of PFS, DMFT, and FT rates was analyzed using PROC SURVEYLOGISTIC, and the results are expressed as odds ratios (OR) with 95% CIs. The significance level was set at *p* < 0.05.

## 3 Results

### 3.1 Trends in the Proportion of Annual PFS Use Among Korean Adolescents (2005–2024)

**FIGURE 1:**
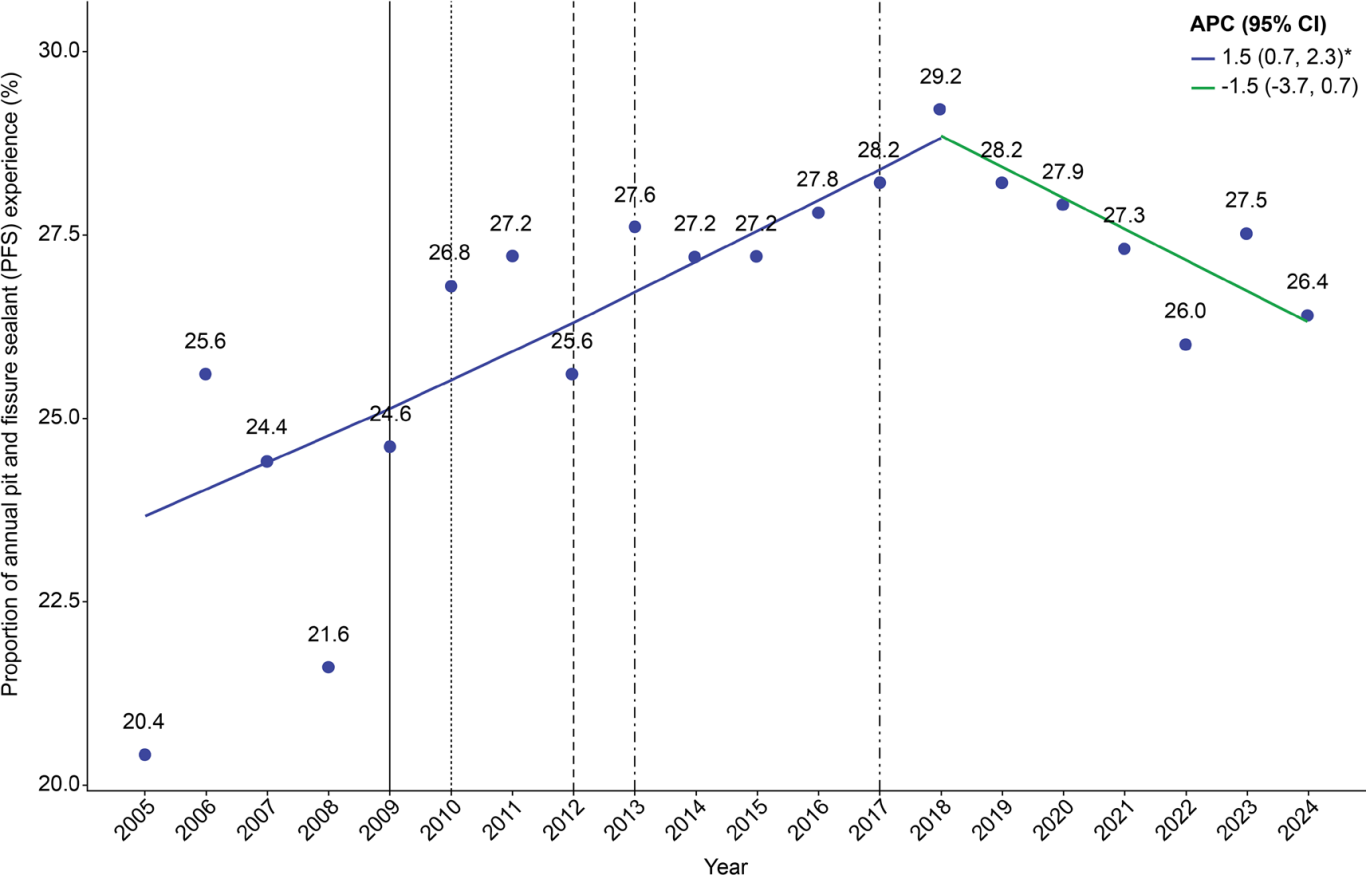
Trends in the proportion of annual pit and fissure sealant (PFS) experience (2005–2024). Fraction of those who received PFS in the last 12 months. Vertical solid line: Dental sealant under insurance coverage (age 6–14, first molar). Vertical dotted line: Dental sealant under insurance coverage (age 6–14, first molar occlusal surface). Vertical dashed line: Dental sealants under insurance coverage (age ≤ 14 years, occlusal surfaces of the first and second molars). Vertical dash‐single dotted line: Dental sealant under insurance coverage (age ≤ 18 years, occlusal surfaces of the first and second molars). Vertical dash‐double dotted line: Dental sealant under insurance coverage (reduction in out‐of‐pocket rate by 10%). APC, Annual percent change, CI, Confidence interval. *Significant at *p* < 0.05. [Color figure can be viewed at wileyonlinelibrary.com]

The APC in the annual proportion of PFS use among Korean adolescents from 2005 to 2024, obtained using joinpoint regression analysis, was as follows: The APC was 1.5% (95% CI: 0.7, 2.3) from 2005 to 2018 (*p* < 0.05) and −1.5% (95% CI: −3.7, 0.7) from 2018 to 2024 (*p* > 0.05; Figure 1).

### 3.2 Trends in DMFT and FT Rates Among Korean Adolescents Obtained Using (2007–2015)

**FIGURE 2:**
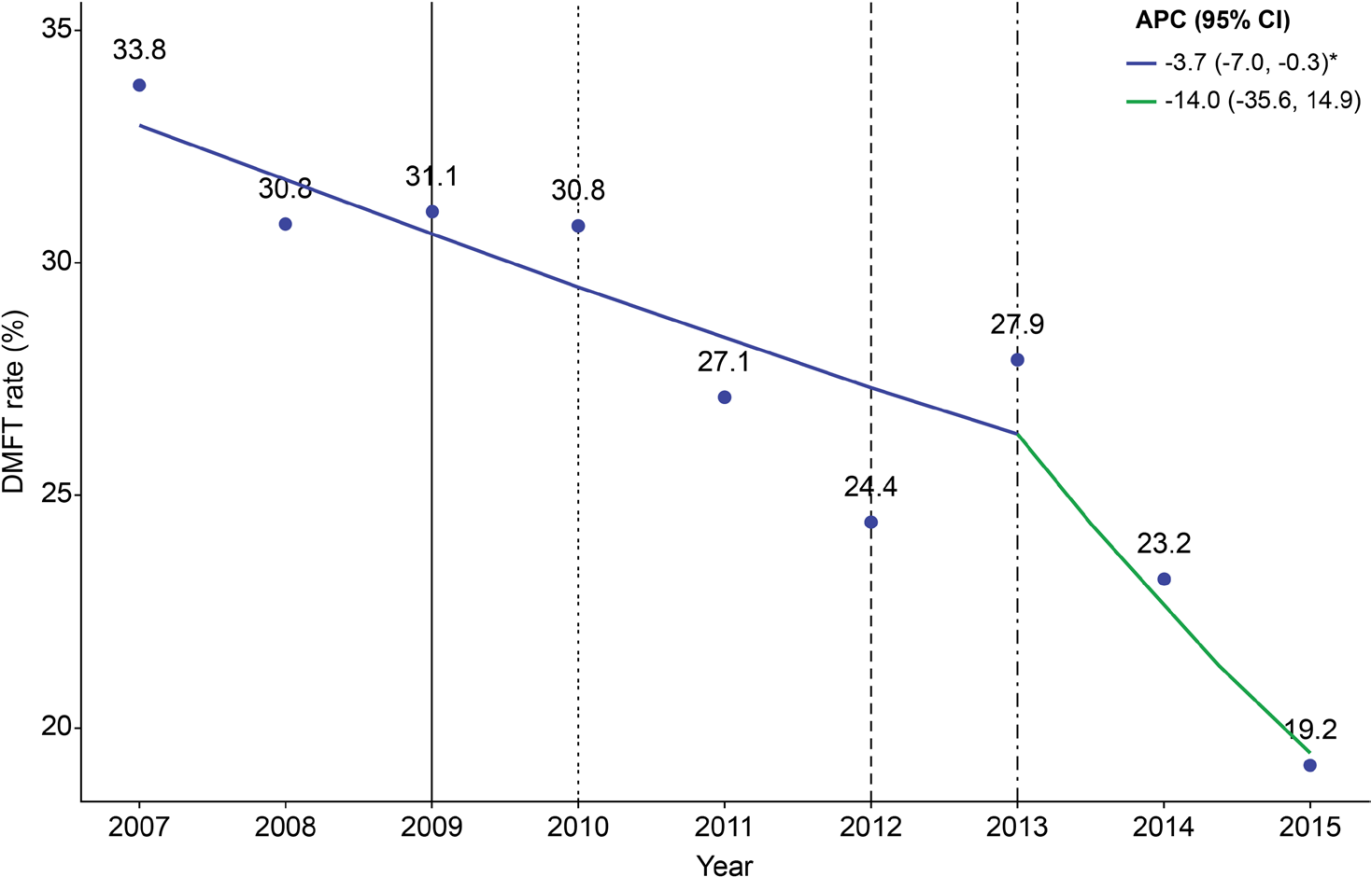
Trends in DMFT rate (2007–2015). Fraction of those who experienced dental caries that occurred in permanent teeth. Vertical solid line: Dental sealant under insurance coverage (age 6–14, first molar). Vertical dotted line: Dental sealant under insurance coverage (age 6–14, first molar occlusal surface). Vertical dashed line: Dental sealants under insurance coverage (age ≤ 14 years, occlusal surfaces of the first and second molars). Vertical dash‐single dotted line: Dental sealant under insurance coverage (age ≤ 18 years, occlusal surfaces of the first and second molars). APC, Annual percent change, CI, Confidence interval. *Significant at *p* < 0.05. [Color figure can be viewed at wileyonlinelibrary.com]

**FIGURE 3:**
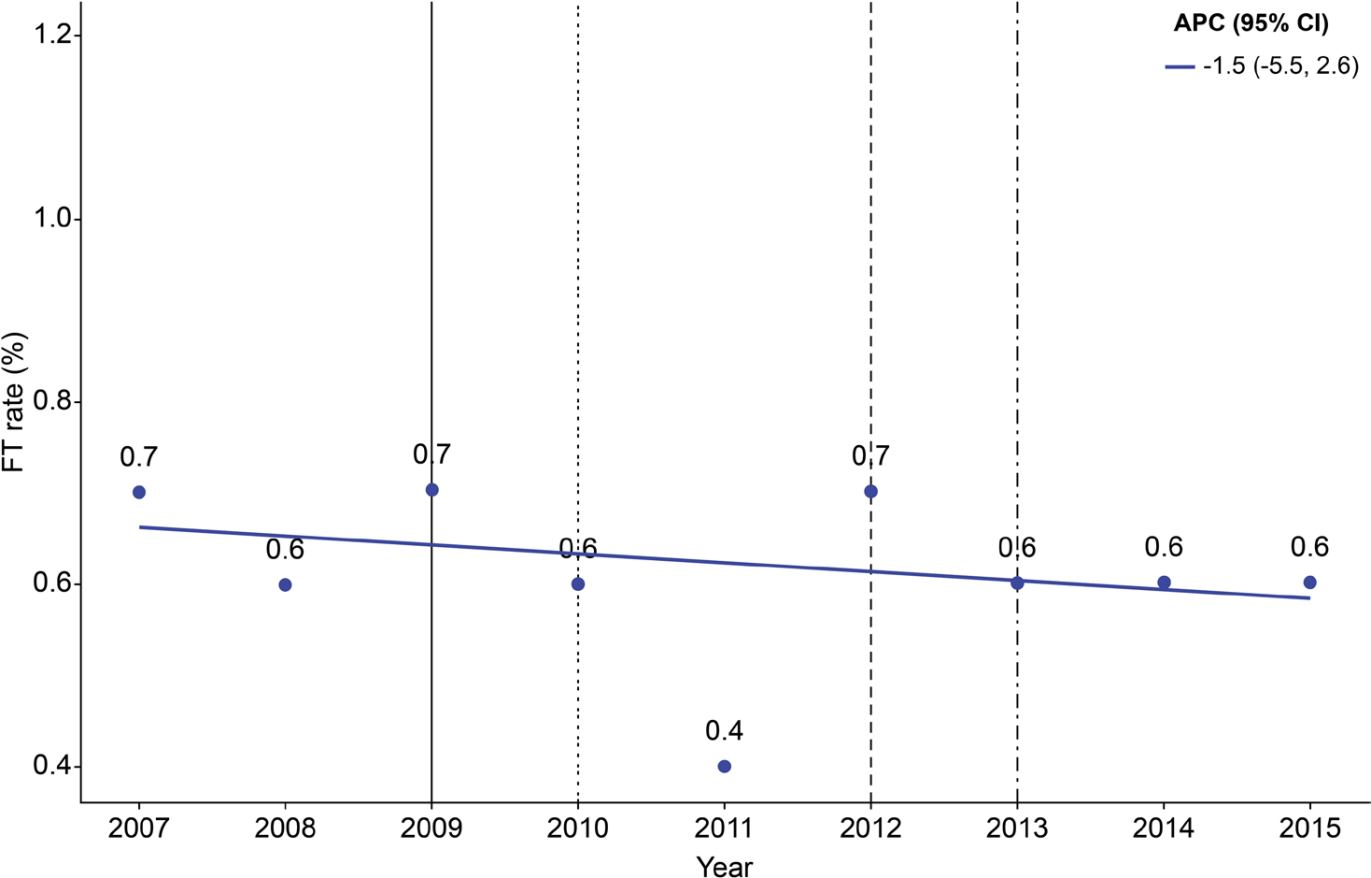
Trends in FT rate (2007–2015). Fraction of those who received filling (restorative) treatment for dental caries in permanent teeth. Vertical solid line: Dental sealant under insurance coverage (age 6–14, first molar). Vertical dotted line: Dental sealant under insurance coverage (age 6–14, first molar occlusal surface). Vertical dashed line: Dental sealants under insurance coverage (age ≤ 14 years, occlusal surfaces of the first and second molars). Vertical dash‐single dotted line: Dental sealant under insurance coverage (age ≤ 18 years, occlusal surfaces of the first and second molars). APC, Annual percent change, CI, Confidence interval. [Color figure can be viewed at wileyonlinelibrary.com]

The results of the analysis of APC in the DMFT rate among Korean adolescents from 2007 to 2015 using joinpoint regression analysis were as follows: The APC was −3.7% (95% CI: −7.0, −0.3) from 2007 to 2013 (*p* < 0.05), and −14.0% (95% CI: −35.6, 14.9) from 2013 to 2015 (*p* > 0.05; Figure 2). Meanwhile, the results of the joinpoint regression analysis indicated that the APC in the FT rate among Korean adolescents was −1.5% (95% CI: −5.5, 2.6) from 2007 to 2015 (*p* > 0.05; Figure 3).

### 3.3 Relationship Between Dental Sealant Insurance Coverage and Proportion of Annual PFS Use Among Korean Adolescents (2005–2024)

The results of the analysis of the relationship between dental sealant insurance coverage and the annual proportion of PFS use among Korean adolescents are presented in Table 1. After adjusting for control variables, the adjusted odds ratios (aORs) for the annual proportion of PFS use at the five time points of dental sealant insurance coverage were 1.20, 1.16, 1.12, 1.12, and 1.06, respectively. Significant increases were observed at all time points (*p* < 0.05).

**TABLE 1:** Relationship between dental sealant insurance coverage and the proportion of annual pit and fissure sealant (PFS) experience among Korean adolescents (2005–2024).

Item	Year	Crude OR (95% CI)	*p*	Adjusted OR (95% CI)	*p*
*Proportion of annual pit and fissure sealant (PFS) experience*					
Dental sealant insurance coverage	2005–2009^a^	ref.	< 0.0001	ref.	< 0.0001
	2010–2024^a^	1.22 (1.20–1.23)		1.20 (1.18–1.22)	
	2005–2010^b^	ref.	< 0.0001	ref.	< 0.0001
	2011–2024^b^	1.19 (1.17–1.20)		1.16 (1.14–1.18)	
	2005–2012^c^	ref.	< 0.0001	ref.	< 0.0001
	2013–2024^c^	1.16 (1.15–1.17)		1.12 (1.11–1.14)	
	2005–2012^d^	ref.	< 0.0001	ref.	< 0.0001
	2013–2024^d^	1.16 (1.15–1.17)		1.12 (1.11–1.14)	
	2005–2017^e^	ref.	< 0.0001	ref.	< 0.0001
	2018–2024^e^	1.10 (1.09–1.12)		1.06 (1.05–1.08)	

*Note*: Values were calculated using PROC SURVEYLOGISTIC. Adjusted for sex, grade, region, academic performance, self‐rated domestic economic situation, father's education, mother's education. The period before policy implementation was used as the reference (ref.) variable.

Abbreviations: CI, Confidence interval; OR, Odds ratio.

^a^ 2009: Insurance coverage for dental sealants for ages 6–14 (first molars).

^b^ 2010: Insurance coverage for dental sealants for ages 6–14 (occlusal surfaces of first molars).

^c^ 2012: Insurance coverage for dental sealants for ages ≤ 14 (occlusal surfaces of first and second molars).

^d^ 2013: Insurance coverage for dental sealants for ages ≤ 18 (occlusal surfaces of first and second molars).

^e^ 2017: Insurance coverage for dental sealants (reduction in out‐of‐pocket rate by 10%).

*Source*: Korean Youth Risk Behavior Survey (KYRBS).

### 3.4 Relationship Between Dental Sealant Insurance Coverage and DMFT and FT Rates Among Korean Adolescents (2007–2015)

The results of the analysis of the relationship between dental sealant insurance coverage and DMFT rate among Korean adolescents are presented in Table 2. After adjusting for control variables, the aORs for DMFT rate at the four time points of dental sealant insurance coverage were 0.72, 0.71, 0.71, and 0.73, respectively. Significant decreases were observed at all four time points (*p* < 0.05).

**TABLE 2:** Relationship between dental sealant insurance coverage and DMFT rate among Korean adolescents (2007–2015).

Item	Year	Crude OR (95% CI)	*p*	Adjusted OR (95% CI)	*p*
*DMFT rate*					
Dental sealant under insurance coverage	2007–2008^a^	ref.	0.0014	ref.	0.0005
	2009–2015^a^	0.75 (0.63–0.89)		0.72 (0.60–0.87)	
	2007–2009^b^	ref.	0.0002	ref.	< 0.0001
	2010–2015^b^	0.74 (0.64–0.87)		0.71 (0.61–0.84)	
	2007–2011^c^	ref.	< 0.0001	ref.	< 0.0001
	2012–2015^c^	0.70 (0.59–0.83)		0.71 (0.60–0.84)	
	2007–2012^d^	ref.	0.0006	ref.	0.0006
	2013–2015^d^	0.73 (0.61–0.87)		0.73 (0.60–0.87)	

*Note*: Values were calculated using PROC SURVEYLOGISTIC. Adjusted for sex, age, region, and household income. The period before policy implementation was used as the reference (ref.) variable.

Abbreviations: CI, confidence interval; OR, odds ratio.

^a^ 2009: Insurance coverage for dental sealants for ages 6–14 (first molars).

^b^ 2010: Insurance coverage for dental sealants for ages 6–14 (occlusal surfaces of first molars).

^c^ 2012: Insurance coverage for dental sealants for ages ≤ 14 (occlusal surfaces of first and second molars).

^d^ 2013: Insurance coverage for dental sealants for ages ≤ 18 (occlusal surfaces of first and second molars).

*Source*: Korean National Health and Nutrition Examination Survey (KNHANES).

The results of the analysis of the relationship between dental sealant insurance coverage and FT rate are listed in Table 3. After adjusting for control variables, the FT rates at the four time points when the policy was implemented were aOR = 0.82, 0.84, 0.85, and 0.73, respectively. Therefore, significant decreases were shown at all four time points (*p* < 0.05).

**TABLE 3:** Relationship between dental sealant insurance coverage and FT rate among Korean adolescents (2007–2015).

Item	Year	Crude OR (95% CI)	*p*	Adjusted OR (95% CI)	*p*
*FT rate*					
Dental sealant under insurance coverage	2007–2008^a^	ref.	0.0620	ref.	0.0406
	2009–2015^a^	0.84 (0.69–1.01)		0.82 (0.68–0.99)	
	2007–2009^b^	ref.	0.0375	ref.	0.0230
	2010–2015^b^	0.85 (0.73–0.99)		0.84 (0.72–0.98)	
	2007–2011^c^	ref.	0.0608	ref.	0.0310
	2012–2015^c^	0.87 (0.75–1.01)		0.85 (0.73–0.99)	
	2007–2012^d^	ref.	0.0003	ref.	0.0001
	2013–2015^d^	0.75 (0.64–0.87)		0.73 (0.62–0.86)	

*Note*: Values were calculated using PROC SURVEYLOGISTIC. Adjusted for gender, age, region, household income. The period before policy implementation was used as the reference (ref.) variable.

Abbreviations: CI, confidence interval; OR, odds ratio.

^a^ 2009: Insurance coverage for dental sealants for ages 6–14 (first molars).

^b^ 2010: Insurance coverage for dental sealants for ages 6–14 (occlusal surfaces of first molars).

^c^ 2012: Insurance coverage for dental sealants for ages ≤ 14 (occlusal surfaces of first and second molars).

^d^ 2013: Insurance coverage for dental sealants for ages ≤ 18 (occlusal surfaces of first and second molars).

*Source*: Korean National Health and Nutrition Examination Survey (KNHANES).

## 4 Discussion

This study examined changes in the annual proportion of PFS use among Korean adolescents after the inclusion of dental sealant under insurance coverage and identified the effects of this policy on the DMFT and FT rates through long‐term analysis.

The results showed that the APC in the proportion of PFS use among adolescents from 2005 to 2024 was 1.5% (95% CI: 0.7, 2.3) from 2005 to 2018 (*p* < 0.05) and −1.5% (95% CI: −3.7, 0.7) from 2018 to 2024 (*p* > 0.05) (Figure 1). Therefore, the proportion of annual PFS use clearly increased during the period when dental sealants were under insurance coverage but declined after 2018. This decline may be due to the coinsurance rate, prompting the Ministry of Health and Welfare to review plans to reduce the rate to 5% [17].

The APC of the DMFT rate among adolescents was −3.7% (95% CI: −7.0, −0.3; *p* < 0.05) from 2007 to 2013, and −14.0% (95% CI: −35.6, 14.9) from 2013 to 2015 (*p* > 0.05; Figure 2). Sohn et al. reported that the prevalence of dental sealant use among Korean adolescents increased from 31.84% in 2007 to 43.69% in 2015, while untreated caries decreased from 13.6% to 6.81% over the same period [18], consistent with the findings of this study. These results indicate that the policy of including dental sealants under insurance coverage positively affected caries prevention among Korean adolescents.

Meanwhile, the APC of the FT rate was −1.5% (95% CI: −5.5, 2.6; *p* > 0.05; Figure 3). This differs from a previous study [19] conducted in an oral health program for elementary school students in Gimje‐si, where the FT rate of the program group (45.19%) was higher than that of the control group (38.04%). This discrepancy may be because the present study analyzed nationwide trends, whereas the previous study focused on a specific, actively managed group region.

Analysis of the annual proportion of PFS use following the five stages of insurance coverage implementation (Table 1) showed significant increases, with aORs of 1.20, 1.16, 1.12, 1.12, and 1.06, respectively (*p* < 0.05). This suggests that the insurance policy had a positive effect on increasing PFS use among Korean adolescents and was effective for preventing dental caries. Similarly, Choi et al. reported a 5.4% increase in PFS from 2008 to 2014 after insurance coverage but noted that the increase was smaller than in the United States Wisconsin Medicaid program (approximately 8%) [15].

In this study, dental sealant insurance coverage significantly reduced the DMFT rate among Korean adolescents at all four time points (aOR = 0.72, 0.71, 0.71, 0.73; *p* < 0.05; Table 2), and the FT rate also significantly decreased (aOR = 0.82, 0.84, 0.85, 0.73, *p* < 0.05; Table 3). Choi et al. similarly reported that the DMFT rate of first molars decreased from 68.4% to 59.3% after the application of National Health Insurance Service Coverage for first molar sealants [12]. These findings are consistent with previous studies showing a significant association between dental sealant application and reduced dental caries in first permanent molars [20].

This study employed complex sample logistic regression analysis to adjust for socio‐demographic and economic covariates that could influence the PFS use and dental caries experience. The findings revealed a significant increase in PFS use among adolescents across all time points, alongside significant declines in DMFT and FT rates following the implementation of PFS insurance coverage. These findings cannot be attributed solely to socio‐demographic and economic factors, as the PFS insurance coverage policy likely played a role in enhancing preventive dental services utilization and improving oral health among adolescents.

This study has several limitations that should be considered when interpreting the findings. First, the generalizability of the results to the entire adolescent population is limited, as it excludes long‐term absentees, students with disabilities, and students with dyslexia. Second, the PFS experience was assessed via self‐reported data, which may be less accurate than oral examinations. Previous research indicates that the self‐report method has a lower accuracy than oral examinations, with a tendency to underreport dental caries and overreport tooth loss and fillings [21]. Among the younger age groups in particular, recall may be inaccurate or influenced by parental reports, leading to potential recall bias. Therefore, the results should be interpreted with caution. Third, this study did not account for factors such as oral health education experience, perceived preventive effects of PFS, toothbrushing and use of oral care products, dietary patterns, free sugar intake, and sugar‐sweetened beverage consumption, that may influence PFS and dental caries experience [22, 23, 24, 25]. Thus, further studies should incorporate these variables and address the limitations of the KYRBS to provide a more comprehensive understanding of the observed associations. However, the study remains important as it objectively analyzed long‐term national trends using representative data and joinpoint regression analysis [26].

In conclusion, in this study, we examined the long‐term trends and policy effects of dental health insurance coverage for PFS on caries experience among Korean adolescents. The annual proportion of PFS use increased until 2018 and then slightly decreased, whereas the DMFT and FT rates showed overall downward trends. Insurance coverage for PFS was significantly associated with increased PFS use and decreased DMFT and FT rates, demonstrating that the policy effectively contributed to the prevention of dental caries among Korean adolescents.

## Funding

The authors have nothing to report.

## Ethics Statement

This study was approved by the Institutional Review Board of Yonsei University Mirae Campus (IRB reference number: 1041849‐202503‐BM‐036‐01).

## Conflicts of Interest

The authors declare no conflicts of interest.

## Supporting information


**Table S1:** Study participants of the Korean Youth Risk Behavior Survey (KYRBS).
**Table S2:**. Survey periods of the Korean Youth Risk Behavior Survey (KYRBS) and the Korean National Health and Nutrition Examination Survey (KNHANES).
**Table S3:** Time of application of pit and fissure sealant (PFS) under insurance coverage and the period during which the raw data were used for analysis.

## Data Availability

Data sharing not applicable to this article as no datasets were generated or analyzed during the current study.
