# Supplementary material for: Trends in Pit and Fissure Sealant Use and Decayed, Missing, and Filled Teeth Rates Among Korean Adolescents After Including Dental Sealants Under Insurance Coverage
Source: J Public Health Dent. 2026 Mar 10;86(2):213–9. doi: 10.1111/jphd.70052 (PMC13241914; doi:10.1111/jphd.70052)
Supplement: Supplementary file 1 — Table S1: Study participants of the Korean Youth Risk Behavior Survey (KYRBS). Table S2:. Survey periods of the Korean Youth Risk Behavior Survey (KYRBS) and the Korean National Health and Nutrition Examination Survey (KNHANES). Table S3: Time of application of pit and fissure sealant (PFS) under insurance coverage and the period during which the raw data were used for analysis. [file JPHD-86-213-s001.docx]

**Supplemental Material Tables**

| **Supplementary Table 1.** Study participants of the Korean Youth Risk Behavior Survey (KYRBS) | | | |
| --- | --- | --- | --- |
| Year | Population | Target Participants (n) | Study Participants (n) |
| 2005 | 3,174,637 | 65,482 | 58,727 |
| 2006 | 3,851,168 | 78,593 | 71,404 |
| 2007 | 3,904,533 | 78,834 | 74,698 |
| 2008 | 3,930,597 | 79,099 | 75,238 |
| 2009 | 3,879,356 | 76,937 | 75,066 |
| 2010 | 3,917,241 | 74,980 | 73,238 |
| 2011 | 3,917,241 | 79,202 | 75,643 |
| 2012 | 3,832,799 | 76,980 | 74,186 |
| 2013 | 3,672,574 | 75,149 | 72,435 |
| 2014 | 3,532,149 | 74,167 | 72,060 |
| 2015 | 3,349,763 | 70,362 | 68,043 |
| 2016 | 3,185,712 | 67,983 | 65,528 |
| 2017 | 3,027,488 | 64,991 | 62,276 |
| 2018 | 2,850,118 | 62,823 | 60,040 |
| 2019 | 2,683,547 | 60,100 | 57,303 |
| 2020 | 2,631,888 | 57,925 | 54,948 |
| 2021 | 2,629,588 | 59,426 | 54,848 |
| 2022 | 2,589,173 | 56,213 | 51,850 |
| 2023 | 2,581,964 | 56,935 | 52,880 |
| 2024 | 2,612,077 | 57,580 | 54,653 |

| **Supplementary Table 2.** Survey periods of the Korean Youth Risk Behavior Survey (KYRBS) and the Korean National Health and Nutrition Examination Survey (KNHANES) | | | | | |
| --- | --- | --- | --- | --- | --- |
| Data Source | Number of times (year) | Survey Period | Data Source | Number of period (year) | Survey Period |
| Korean Youth Risk Behavior Survey (KYRBS) | 1^st^ (2005) | 10.10–11.30 | Korean National Health and Nutrition Examination Survey (KNHANES) | 4^th^ (2007) | 07.01–12.31 |
|  | 2^nd^ (2006) | 09.01–10.30 |  | 4^th^ (2008–2009) | 01.01–12.31 |
|  | 3^rd^ (2007) | 09.01–11.03 |  | 5^th^ (2010–2012) | 01.01–12.31 |
|  | 4^th^ (2008) | 09.01–10.24 |  | 6^th^ (2013–2015) | 01.01–12.31 |
|  | 5^th^ (2009) | 09.01–11.20 |  |  |  |
|  | 6^th^ (2010) | 09.01–10.24 |  |  |  |
|  | 7^th^ (2011) | 09.01–10.17 |  |  |  |
|  | 8^th^ (2012) | 06.01–07.17 |  |  |  |
|  | 9^th^ (2013) | 06.01–07.18 |  |  |  |
|  | 10^th^ (2014) | 06.02–07.18 |  |  |  |
|  | 11^th^ (2015) | 06.01–07.28 |  |  |  |
|  | 12^th^ (2016) | 06.01–07.21 |  |  |  |
|  | 13^th^ (2017) | 06.01–07.18 |  |  |  |
|  | 14^th^ (2018) | 06.04–07.19 |  |  |  |
|  | 15^th^ (2019) | 06.03–07.12 |  |  |  |
|  | 16^th^ (2020) | 08.03–11.13 |  |  |  |
|  | 17^th^ (2021) | 08.30–11.11 |  |  |  |
|  | 18^th^ (2022) | 08.29–10.25 |  |  |  |
|  | 19^th^ (2023) | 08.28–10.19 |  |  |  |
|  | 20^th^ (2024) | 06.10–07.06 |  |  |  |

| **Supplementary Table 3.** Time of application of pit and fissure sealant (PFS) under insurance coverage and the period during which the raw data were used for analysis | | | | | |
| --- | --- | --- | --- | --- | --- |
| Item | Time of application | Korean Youth Risk Behavior Survey (KYRBS) | | Korean National Health and Nutrition Examination Survey (KNHANES) | |
|  |  | Period before application | Period after application | Period before application | Period after application |
| Dental sealant under insurance coverage | 2009. 12. 01 | 2005–2009 | 2010–2024 | 2007–2008 | 2009–2015 |
|  | 2010. 12. 01 | 2005–2010 | 2011–2024 | 2007–2009 | 2010–2015 |
|  | 2012. 10. 01 | 2005–2012 | 2013–2024 | 2007–2011 | 2012–2015 |
|  | 2013. 05. 06 | 2005–2012 | 2013–2024 | 2007–2012 | 2013–2015 |
|  | 2017. 10. 01 | 2005–2017 | 2018–2024 | NA | NA |

NA: Not Applicable
